# Synaptic boutons are smaller in chandelier cell cartridges in autism

**DOI:** 10.1371/journal.pone.0281477

**Published:** 2023-04-25

**Authors:** Tiffany Hong, Erin McBride, Brett D. Dufour, Carmen Falcone, Mai Doan, Stephen G. Noctor, Verónica Martínez-Cerdeño

**Affiliations:** 1 Department of Pathology and Laboratory Medicine, Institute for Pediatric Regenerative Medicine and Shriners Hospitals for Children of Northern California, UC Davis School of Medicine, Sacramento, CA, United States of America; 2 Department of Psychiatry and Behavioral Science, UC Davis School of Medicine, Sacramento, CA, United States of America; 3 MIND Institute, UC Davis Medical Center, Sacramento, CA, United States of America; Nanjing University, CHINA

## Abstract

Chandelier (Ch) cells are cortical interneurons with axon terminal structures known as cartridges that synapse on the axon initial segment of excitatory pyramidal neurons. Previous studies indicate that the number of Ch cells is decreased in autism, and that GABA receptors are decreased in the Ch cell synaptic target in the prefrontal cortex. To further identify Ch cell alterations, we examined whether the length of cartridges, and the number, density, and size of Ch cell synaptic boutons, differed in the prefrontal cortex of cases with autism versus control cases. We collected samples of postmortem human prefrontal cortex (Brodmann Area (BA) 9, 46, and 47) from 20 cases with autism and 20 age- and sex-matched control cases. Ch cells were labeled using an antibody against parvalbumin, a marker that labeles soma, cartridges, and synaptic boutons. We found no significant difference in the average length of cartridges, or in the total number or density of boutons in control subjects vs. subjects with autism. However, we found a significant decrease in the size of Ch cell boutons in those with autism. The reduced size of Ch cell boutons may result in reduced inhibitory signal transmission and impact the balance of excitation to inhibition in the prefrontal cortex in autism.

## Introduction

**Fig 1:**
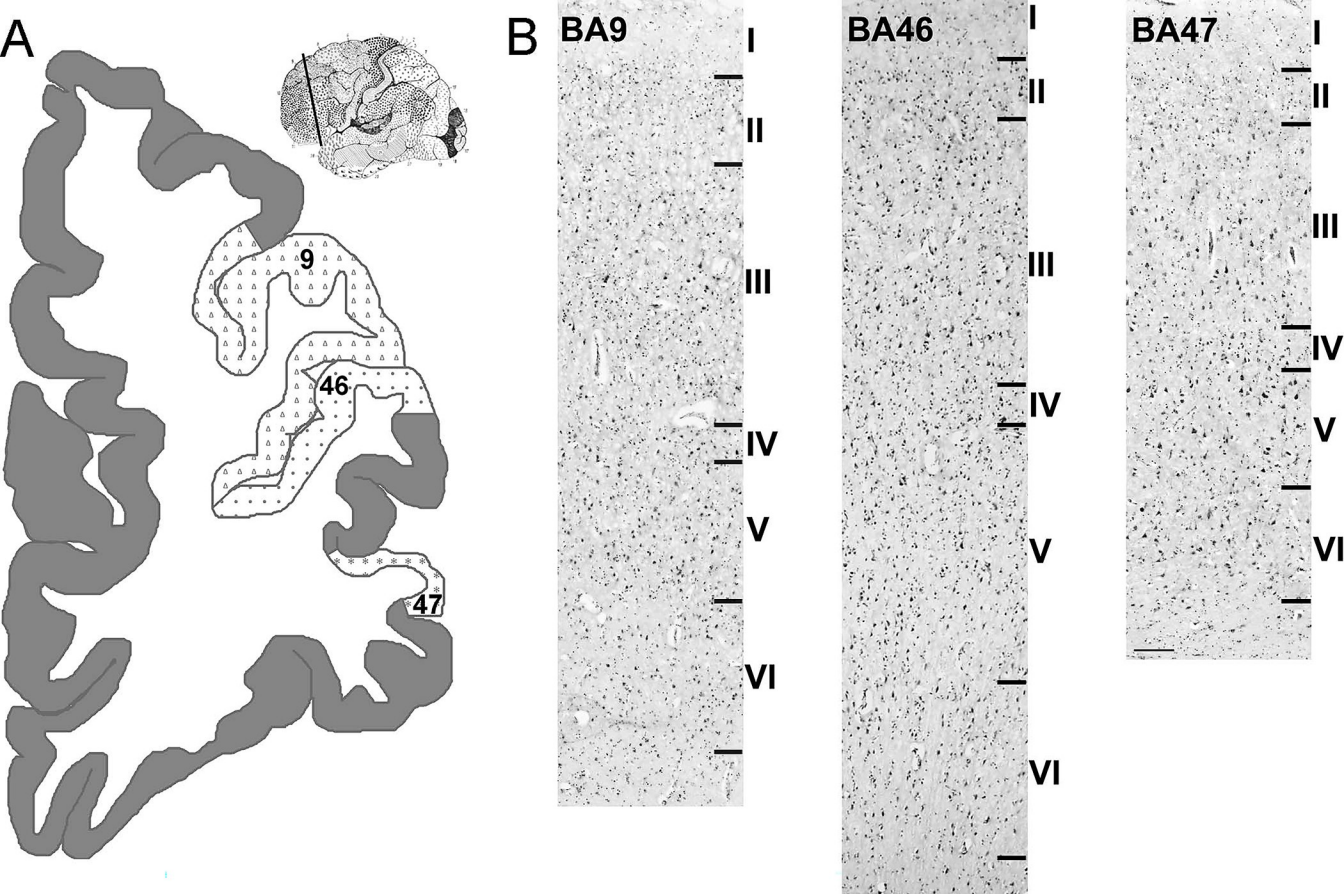
Cortical areas of interest stained with Nissl. Blocks of prefrontal cortical tissue containing BA9, BA46, and BA47 were isolated based on Brodmann and von Economo analysis. A. Coronal section of cerebral cortex from a left hemisphere of Brodmann areas BA9, BA46, and BA47. Adjacent area BA45 is marked for reference. B-D. Nissl-stained sections of (B) BA9, (C) BA46, and (D) BA47. Short horizontal lines in B-D denote layer boundaries. Scale bar in A: 0.5cm, scale bar in D (B-D): 200μm.

Autism is a neurodevelopmental disorder characterized by impaired social communication and repetitive behaviors [1]. The neuropathology of autism has been associated with an imbalance between excitation and inhibition in areas of the cerebral cortex concerned with cognition, language, and social communication [2]. The number of chandelier (Ch) cells, an inhibitory interneuron subtype that expresses the calcium-sequestering protein parvalbumin (PV), is decreased in prefrontal cortex Brodmann Areas (BA) 9, 46, and 47 in autism [3–5]. Dorsolateral prefrontal cortex areas BA9 and BA46 modulate attention and behavior [6], while BA47 in the ventrolateral prefrontal cortex plays a role in language processing [7]. Ch cells are fast-spiking gamma-aminobutyric acid (GABA)ergic interneurons characterized by vertically oriented axon terminals called cartridges, which are rows of synaptic boutons linked by a cytoplasmic bridge [8–10]. Ch cells modulate excitatory pyramidal neuron activity via synapses on pyramidal neuron axon initial segments (AIS). It is feasible that the altered numbers of Ch cells previously shown could contribute to an electrical imbalance in autism, and the remaining Ch cells may compensate to some extent through altered synaptic activity. Each individual Ch cell contacts up to 50% of all pyramidal neurons within the area traversed by its axonal arbor [11,12]. Innervated pyramidal neurons are not positioned at random, but show a clustered distribution; some pockets of pyramidal neurons within a Ch cell axonal tree are all innervated, while others receive little or no innervation [12]. A single pyramidal neuron receives innervation from one to four Ch cells, and a single Ch cell innervates hundreds of pyramidal neurons, highlighting the importance of Ch cells in cortical circuit regulation [11,13–16]. Thus, the alteration in Ch cell number may contribute to an excitation / inhibition imbalance in autism. In addition, GABA_A_Rα2 (GABA_A_ receptor subunit α2) is decreased in the pyramidal neuron AIS in the prefrontal cortical areas BA9 and BA47 in autism [17]. This decrease could be a response to a change of synaptic connection number and/or synaptic connection strength via synaptic boutons in Ch cells. To better understand the Ch cell properties in autism, we quantified bouton number and size, and cartridge length of Ch cells in the prefrontal cortex (BA9, BA46 and BA47) in postmortem human tissue from cases with autism compared to age- and sex-matched cases without neurological disorders (Table 1, Fig 1).

**Table 1:** Subjects included in this study. Columns include: Subject ID, diagnosis, sex, age, postmortem interval (PMI), time in formalin, and cause of death. CT = Control. AU = Autism. NK = not known. One subject (ID: 4305) presented with seizures. Control subjects were defined as free of neurological disorders, including autism, based on medical records and information gathered at the time of death from next of kin.

Case ID	Diagnosis	Sex	Age (years)	PMI (hours)	Time in formalin (months)	Cause of Death
UCD13AP86	CT	M	6	44.3	64	NK
4203	CT	M	7	24	164	Respiratory insufficiency
4337	CT	M	8	16	97	Blunt force
210	CT	M	10	18	278	Myocarditis
5834	CT	M	14	38	26	Cardiac arrhythmia
AN07444	CT	M	17	30.8	74	Asphyxia
AN00544	CT	M	17	28.9	NK	NK
5893	CT	M	19	19	21	Dilated cardiomegaly
5958	CT	M	22	24	13	Dilated cardiomegaly
AN01891	CT	M	24	35	86	NK
UCD1602	CT	M	26	35.7	28	NK
UCD1505	CT	M	26	>72	47	Renal disease
AN19760	CT	M	28	23.3	NK	NK
AN12137	CT	M	31	32.9	NK	Asphyxia
AN15566	CT	F	32	28.9	NK	NK
UCD1510	CT	M	35	>72	39	NK
AN05475	CT	M	39	NK	123	Cardiac arrest
AN17868	CT	M	46	18.8	NK	Cardiac arrest
AN19442	CT	M	50	20.4	NK	NK
AN13295	CT	M	56	22.1	NK	NK
AN03221	AU	M	7	11.4	123	Drowning
5144	AU	M	7	3	109	Cancer
AN01293	AU	M	9	4.4	120	Cardiac arrest
4305	AU	M	12	13	119	Serotonin syndrome
4899	AU	M	14	9	128	Drowning
AN00394	AU	M	14	10.3	197	Cardiac arrest
5403	AU	M	16	35	82	Cardiac arrhythmia
4269	AU	M	19	45	135	Meningitis
4999	AU	M	20	14	111	Cardiac arrhythmia
AN00764	AU	M	20	23.7	167	Accident
5176	AU	M	22	18	106	Subdural hemorrhage
5574	AU	M	23	14	56	Pneumonia
AN00493	AU	M	27	8.3	171	Drowning
AN09412	AU	M	29	38	42	NK
AN18892	AU	M	31	>72	177	Gun shot
5027	AU	M	37	26	119	Bowel obstruction
1575	AU	F	40	24	136	Complications of diabetes
AN06746	AU	M	44	30.8	216	Cardiac arrest
5137	AU	M	51	72	107	Pneumonia
AN01093	AU	M	56	NK	190	NK

Cases included in this study. Columns include subject ID, diagnosis, sex, age, postmortem interval (PMI), time in formalin, and cause of death. CT = Control. AU = Autism. NK = not known.

## Materials and methods

### Samples

We obtained postmortem human tissue samples from the Autism Tissue Program (ATP) (predecessor to Autism BrainNet) and the NIH NeuroBioBank. Brain banks used the Autism Diagnostic Interview-Revised (ADI-R) to confirm diagnosis. Control (CT) cases were free of neurological disorders, based on medical records and information gathered at the time of death from next of kin. This study includes twenty cases with autism and twenty age- and sex-matched control cases. Of those 38 were males (n = 19 CT + 19 AU) and 2 females (n = 1 CT + 1 AU), (Table 1). One subject with autism presented with seizures (Table 1). The average age of control cases was 24.9 years (from 7 to 56 years), and of cases with autism was 25.6 years (from 6 to 56 years). There was no difference in PMI between control and autism groups (31.8 hours for CT and 24.8 for AU, p > 0.05). For causes of death see Table 1. Blocks of Brodmann areas BA9, BA46 and BA47 were isolated based on Brodmann anatomy [4]. Tissue blocks were fixed in 10% buffered formalin and cryoprotected in a 30% sucrose. Tissue was embedded in Optimum Cutting Temperature (OCT) compound and frozen. 14μm-thick slide-mounted sections were cut in a cryostat and stored at -80°C until use. Occasionally, tissue for a given case was unsuitable for analysis, including: BA9 from subject UCD13AP86; BA46 from cases 210, 4899, 4999, and AN00764; and BA47 from cases 4337, 210, 4899 and 5574; no data was excluded on the basis of age, sex, or PMI. One section per block was Nissl-stained to confirm cortical areas based on von Economo histology (Fig 1), (Hashemi et al., 2017), and adjacent sections were used for immunostaining.

### Immunostaining

Slide mounted tissue sections were enzymatically immunostained with an antibody against parvalbumin. Briefly, tissue was treated with chloroform:100% ethanol at 1:1 followed by sequential immersion in 100%, 96%, 90%, 70% and 50% EtOH, then diH_2_0. Antigen retrieval was performed by exposing the tissue to 110°C in 1x Diva Decloaker (Biocare Medical) for 8 min., and slides were washed (all washes consisted of TBS twice followed by TBS + 0.05% tween once). Endogenous peroxidase blocking was performed with 3% H_2_O_2_. The slides were blocked with tris buffered saline (TBS) + 10% normal donkey serum (NDS) + 0.3% triton for 1 hour at room temperature, and then treated with an avidin-biotin blocking kit (Vector Labs). Primary antibody solution (rabbit anti-parvalbumin antibody, Abcam, 1:500) was added to each slide for 24 hours at 4˚C with Parafilm coverslips in a dark humidified box. Slides were then washed and incubated with the secondary antibody (biotinylated donkey anti-rabbit IgG, Jackson, 1:150) for 1 hour, then washed and incubated with ABC solution (Vector Labs) for 2 hours in a dark, humidified box. After washing, slides were developed with DAB substrate (brown; Vector Labs) for 1 minute, then washed again. Tissue was dehydrated through sequential immersion in 50%, 70%, 90%, 96%, and 100% EtOH for 3 minutes each, then cleared in Xylene for 6 minutes and coverslipped with Permount mounting medium.

### Antibody characterization

Anti-Parvalbumin antibody: *Immunogen*: Full length native protein (purified) corresponding to Rat Parvalbumin. Purified parvalbumin from rat skeletal muscle; *Sequence similarities*: Belongs to the parvalbumin family. Contains 2 EF-hand domains; *Host*: Rabbit; *Isotype*: IgG; *Antibody type*: polyclonal; *Manufacturer and Catalogue#*: Abcam ab11427; *Concentration*: 1:500; *Additional information*: Reacts with mouse, rat, chicken, human, and sea urchin, and predicted to work with gerbil and common marmoset; *RRID*: AB_298032.

### Imaging, quantification, and statistics

**Fig 2:**
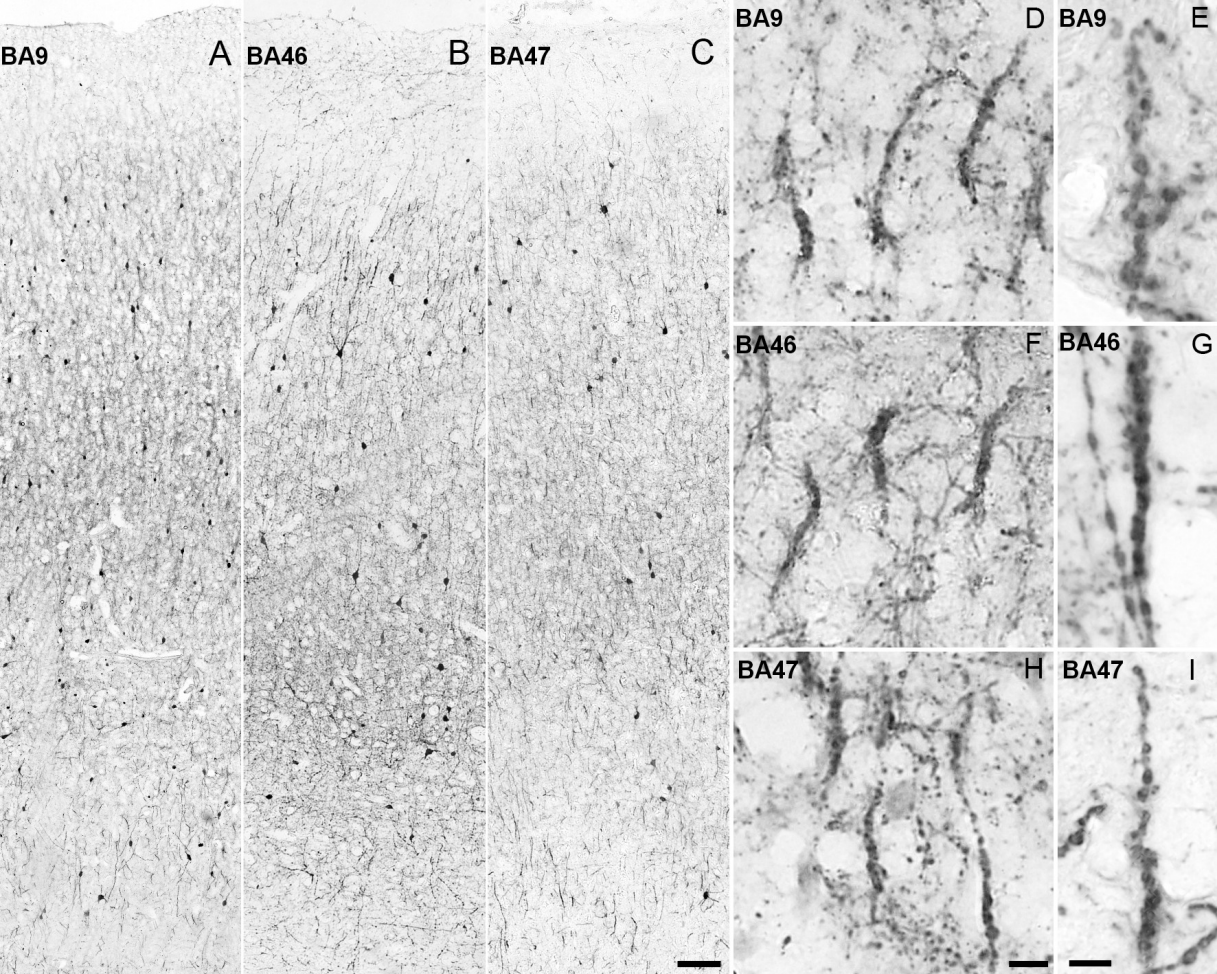
Ch cell cartridges stained with an antibody against PV in Brodmann areas BA9 (A,D,E), BA46 (B,F,G), and BA47 (C,H,I) at low and high magnification. Scale bar in A-C: 500 μm; D,F,H: 10 μm; E,G, I: 5 μm.

PV immunoreactivity in Ch cell cartridges across prefrontal areas BA9, BA46 and BA47 was located most clearly in layers II/III and layer V (Fig 2). The PV staining pattern was consistent across our cases, and qualitative analysis did not reveal a noticeable difference in the Ch cartridge expression of PV between autism and control cases.

**Fig 3:**
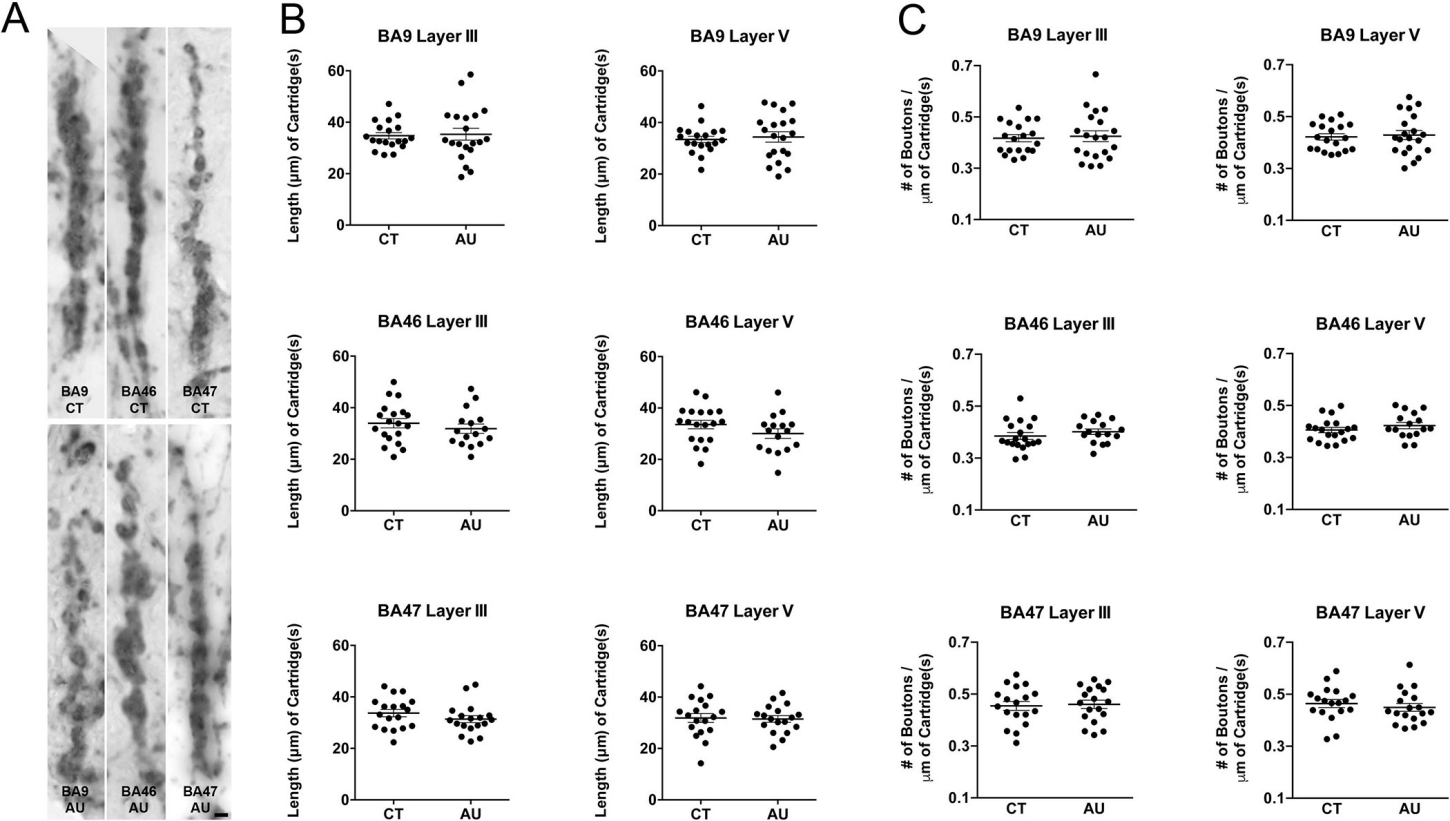
A. Ch cell cartridges stained with PV in control and ASD prefrontal cortex. B-C: The length of cartridges (B) and the number of boutons per μm of cartridge (C) are similar in ASD and control cases, in both supra- and infragranular layers. Scale bar in A: 2 μm.

We captured brightfield images using the 100x objective of an Olympus microscope (BX61). Five cartridges per layer, per Brodmann Area, per case were captured, and cartridges were chosen based on quality and homogeneity of staining, cartridge completeness, and clearly distinguishable boutons. We then measured the length of the cartridges using the ImageJ plugin NeuronJ. To quantify the number of synaptic boutons (Fig 3), each cartridge was analyzed separately by two researchers, each blinded to the diagnoses, and the two duplicate counts were then averaged together. To measure bouton size, individual boutons were first identified within a cartridge by imposing a circularity threshold, which outlined round/oval shapes within an image. Subsequently, if any boutons were missed or unclearly marked, or if any other structure was erroneously identified as a bouton, borders were manually corrected using the selection brush tool until each bouton within the cartridge was distinctly delineated. The size of each bouton was then quantified using Feret’s diameter (ImageJ).

Data collected from the supragranular (layer III) and infragranular (layer V) layers (5 cartridges per layer) were analyzed for each area (BA9, BA46, BA47). Data for a given layer of a given case was comprised of the average values from all cartridges analyzed within that layer of that case. The goal of the statistical analysis was to compare cartridge synaptic bouton number and density, bouton size, and cartridge length between autism and control cases, and to assess the relationship between anatomical parameters and other patient/sample characteristics (such as age, PMI, and time in formalin). We performed a repeated measures mixed linear regression model analysis that accounted for the nested (diagnosis within subject) and crossed factors (counts within layers within regions) and avoids pseudo replication.

## Results

We labeled Ch cells with an antibody against parvalbumin, measured cartridge lengths, and quantified the number and size of synaptic boutons, in prefrontal cortex (BA9, BA46, and BA47) in tissue obtained from 20 cases with autism (AU) and 20 age- and sex-matched control (CT) cases (Table 1), Superposed cartridges or individual cartridges overlaid on a single AIS are referred to as “cartridges”.

We measured the length of each cartridge using the ImageJ plugin NeuronJ. We did not find a difference in the length of cartridges in either supragranular or infragranular layers in cases with autism compared with control cases (all p > 0.05). In BA9, average cartridge length in layer III was 35.69 ± 1.2 μm for control and 40.37 ± 2.6 μm for autism cases, and in layer V cartridge length was 32.85 ± 0.7 μm for control and 40.62 ± 1.8 μm for autism cases. In BA46, average cartridge length in layer III was 34.20 ± 1.2 μm for control and 34.33 ± 2.0 μm for autism cases, and in layer V cartridge length was 33.25 ± 1.6 μm for control and 35.37 ± 1.6 μm for autism cases. In BA47, average cartridge length in layer III was 34.18 ± 1.5 μm for control and 34.82 ± 2.3 μm for autism cases, and in layer V cartridge length was 34.75 ± 1.6 μm for control and 29.98 ± 2.0 μm for autism cases (Fig 3).

In addition, we found no significant difference in the total number or density of Ch cell boutons between control and autism cases in either supragranular or infragranular layers of any area analyzed (all p > 0.05). In BA9, the total number of boutons in layer III was 14.31 ± 0.5 for control and 14.03 ± 0.6 for autism cases, and in layer V the total number of boutons was 13.68 ± 0.5 for control and 14.04 ± 0.7 for autism cases. In BA46, the total number of boutons in layer III was 12.67 ± 0.6 for control and 12.65 ± 0.8 for autism cases, and in layer V the total number of boutons was 13.39 ± 0.6 for control and 12.29 ± 0.6 for autism cases. In BA47, the total number of boutons in layer III was 14.67 ± 0.6 for control and 13.80 ± 0.5 for autism cases, and in layer V the total number of boutons was 14.05 ± 0.6 for control and 13.84 ± 0.5 for autism cases (Fig 3).

In BA9, the density of cartridges in layer III was 0.42 ± 0.01 boutons/μm of cartridge in control and 0.42 ± 0.02 boutons/μm of cartridge in autism cases, and in layer V the density of cartridges was 0.42 ± 0.01 boutons/μm of cartridge in control and 0.43 ± 0.02 boutons/μm of cartridge in autism cases. In BA46, the density of cartridges in layer III was 0.38 ± 0.01 boutons/μm of cartridge in control and 0.40 ± 0.01 boutons/μm of cartridge in autism cases, and in layer V the density of cartridges was 0.41 ± 0.01 boutons/μm of cartridge in control and 0.42 ± 0.01 boutons/μm of cartridge in autism cases. In BA47, the density of cartridges in layer III was 0.45 ± 0.02 boutons/μm of cartridge in control and 0.46 ± 0.02 boutons/μm of cartridge in autism cases, and in layer V the density of cartridges was 0.46 ± 0.02 boutons/μm of cartridge in control and 0.45 ± 0.02 boutons/μm of cartridge in autism cases (Fig 3).

**Fig 4:**
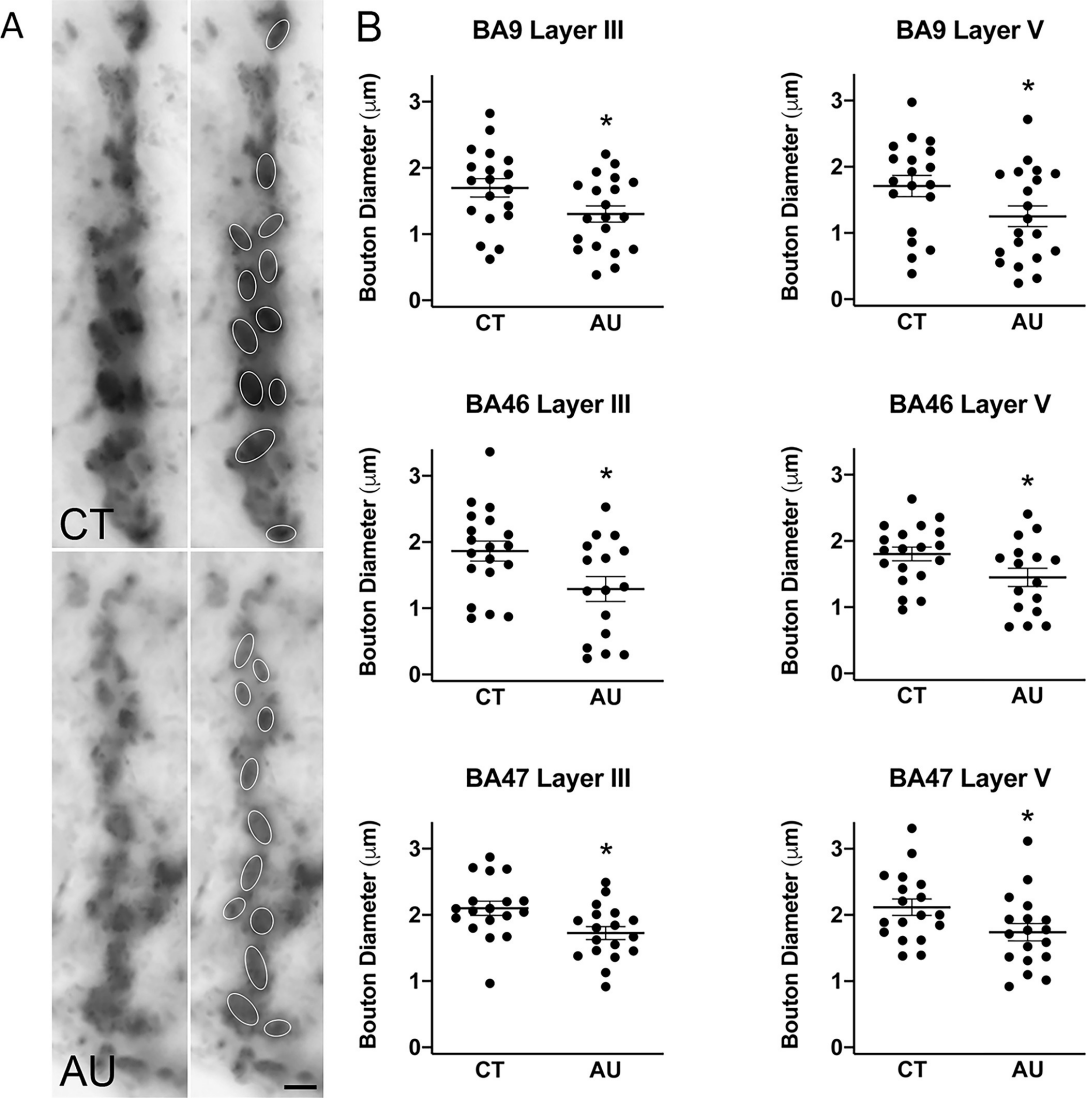
A. Ch cell cartridges stained with PV in control and ASD prefrontal cortex. Boutons are delineated in white. B. Bouton size is decreased in ASD when compared to controls in supra- and infragranular layers. Asterisk denotes statistically significant difference. Scale bar in A: 2 μm.

We next measured the size of each bouton using the ImageJ Feret’s diameter protocol and found a decreased terminal bouton diameter per cartridge in autism compared to control cases, in all layers and all areas (all p < 0.05). In BA9, average bouton diameter in layer III was 1.70 ± 0.1 μm for control and 1.30 ± 0.1 μm for autism cases, and in layer V average bouton diameter was 1.71 ± 0.1 μm for control and 1.25 ± 0.1 μm for autism cases (a 23.5% and 26.9%, decrease, respectively). In BA46, the average bouton diameter in layer III was 1.86 ± 0.1 μm for control and 1.29 ± 0.1 μm for autism cases, and in layer V average bouton diameter was 1.80 ± 0.1 μm for control and 1.45 ± 0.1 μm for autism cases (a 30.6% and 19.4% decrease, respectively). In BA47, average bouton diameter in layer III was 2.10 ± 0.1 μm for control and 1.73 ±0.1 μm for autism cases, and in layer V average bouton diameter was 2.12 ± 0.1 μm for control and 1.74 ± 0.1 μm for autism cases (a 17.6% and 17.9% decrease, respectively), (Fig 4).

Statistical analysis showed no significant influence of covariates, including age, PMI, or time in formalin, on bouton number, density, size, or cartridge length in any of the areas or cortical layers analyzed (p > 0.05). There was not a significant effect of layer, or any of its interactions (p > 0.10).

Overall, we found no significant difference in Ch cell bouton number or density and no significant difference in the length of cartridges in either supragranular or infragranular layers in the prefrontal cortex in cases with autism when compared to control cases. However, we found a decrease in the bouton size in Ch cell cartridges in autism when compared to control cases, in all layers and areas analyzed.

## Discussion

Chandelier cell (Ch) axon terminals, termed cartridges, are unique structures consisting of synaptic boutons joined by a cytoplasmic bridge and oriented perpendicular to the cortical surface in alignment with the axon initial segment (AIS) of pyramidal cells [18]. A single pyramidal neuron receives innervation from one to four Ch cells, with Ch cell cartridges overlapping over each pyramidal neuron AIS. Ch cell cartridges have been reported in the cerebral cortex of various mammalian species [19] and can be detected with Golgi staining or single cell labeling techniques. For example, in utero electroporation of Ch cell progenitor cells during prenatal development to introduce enhanced green fluorescence protein (EGFP) [20], or using an Nkx2.1-Cre::MADM transgenic mouse that expresses EGFP in a subset of neocortical interneurons including Ch cells [11], allows for single cell analysis. In human tissue, cartridges can be detected using antibodies against proteins localized to the cartridges, such as PV, GAT1, GAD67, or PSNCAM [5,21–24], but these methods do not permit discernment of cartridges originating from a distinct cell, but rather a combination of all superposed cartridges innervating a single pyramidal AIS. Superposed cartridges are of two types, referred to as “simple” or “complex”, and are differentiated by their size and by the density of axonal boutons [25,26]. In primates, superposed simple cartridges are composed of one or two individual cartridges, each consisting of three to five boutons. By contrast, superposed complex cartridges are tight cylinder-like structures comprised of multiple individual cartridges [18]. Ch cells are particularly complex in human, with larger and morphologically more elaborate cartridges compared to those in mice [27]. Here, we refer to superposed cartridges innervating a single pyramidal AIS in human as “cartridges”.

In the mouse neocortex, the average length of cartridges is 22.2 ± 6 μm [20], and we found that the average length of cartridges in the human prefrontal cortex is approximately 35 μm, suggesting that Ch cell cartridges are longer in humans than in mice. This may reflect an increased complexity of Ch cells in human, as previously shown for other cell types [28–30], and perhaps may also correlate with longer pyramidal axon initial segment in human compared to mice. The number of Ch cell boutons per cartridge varies by brain region, species, and pyramidal cell size [18,31–34]. Ch cell cartridges contains about 3–5 boutons in the mouse neocortex and up to 15 boutons in the sensory-motor cortex of monkeys [11,18,26,35–37]. Our data agrees with previous non-human primate data, showing an average of 13.5 boutons per cartridge in the human prefrontal cortex. The diameter of Ch cell synaptic boutons in the mouse neocortex is 1.4–1.6 μm [20], and we found that Ch boutons are an average of 1.7–2.1 depending on the cortical area. This indicates that Ch cell boutons are bigger in human than in mice, again reflecting the increased complexity of Ch cells in human. We examined and measured Ch cell cartridges and bouton morphology in the human prefrontal cortex for potential alterations that could unveil neuropathological processes in the cortical GABAergic system in autism.

### The length of Ch cell cartridges is not changed in autism

The Ch cell cartridge consists of a string of synaptic boutons connected by a cytoplasmic bridge, and each cartridge is aligned adjacent to the pyramidal cell AIS that innervates (Gallo et al. 2020). A decreased cartridge length in autism could result in fewer synaptic boutons per cartridge and thus fewer synaptic sites, potentially interfering with the Ch cell inhibitory signaling ability. However, we found no difference in Ch cell cartridge length between control and autism cases, with cartridges measuring an average of 35 μm in length across cortical layers, brain regions, and diagnostic categories. It is important to note that the length measured may represent the length of overlapping superposed cartridges innervating the same pyramidal neuron AIS. Our results suggest that while the number of Ch cells and the Ch cell cartridge density are both decreased in the prefrontal cortex in autism [3–5], the maximum length of cartridges on existing Ch cells is not affected and thus is likely not a factor contributing to the decreased GABA_A_ receptor subunit α2 present in the pyramidal AIS in some prefrontal cortical areas in autism [17].

### The number of Ch cell synaptic boutons is not changed in autism

Boutons are axon terminal structures containing sites of synaptic junctions. They are presynaptic points of neurotransmitter storage and release to facilitate cell communication [38]. Boutons contain the active zones where synaptic vesicles dock. Vesicle exocytosis is stimulated through Ca2+ signaling, releasing signaling molecules into the synapse where they are taken up at the adjacent postsynaptic density [39]. Within the boutons, synaptic vesicles undergo cyclic trafficking comprised of exo- and endocytosis in the course of neurotransmitter packaging and release [39], the dynamics of which may be influenced by activity-dependent plasticity [40]. Cell bouton number is linked to Ch cell firing potential and its ability to regulate pyramidal neuron activity [41–43]. Approximately four Ch cells innervate each pyramidal AIS [11,16], and each Ch cell cartridge forms three to eight synapses on the AIS [11,26,35–37]. Bouton number is proportional to the number of synapses [44]. The number and distribution pattern of boutons varies by species, developmental stage, cortical region, and the functional state of cells. For example, the mean number of Ch cell boutons per pyramidal neuron AIS decreases 32% in monkey prefrontal cortex from 3 months of age to adulthood [16]. Additionally, the pattern of bouton distribution is correlated with the size of the pyramidal AIS [18,31–34,45]. In general, synaptic boutons tend to distribute homogeneously with random intervals along the axon and increase in number with axon length, independent of the distance from the soma or the length between branching nodes [44]. Bouton spread facilitates the optimal contact with the target. Along with small basket and double-bouquet cells, Ch cells have among the lowest proportion of boutons farther than 200 μm from the soma, demonstrating a highly compact bouton field, as well as a unique pattern of bouton clustering along axon cartridges [44].

We did not find a difference in the number or density of Ch cell terminal boutons per cartridge in autism vs. control cases. Our results may indicate that the synaptic input to a single pyramidal cell is conserved in autism. However, the number of Ch cells is decreased by approximately half in the prefrontal cortex in autism, suggesting an increase in the number of innervating cartridges either from one or several adjacent Ch cells to a single pyramidal neuron AIS, or that we quantified cartridges from areas of unchanged Ch cell density. As the parvalbumin immunostain does not distinguish individual cartridges, further study would be necessary to elucidate whether bouton density per individual cartridge is also unchanged, and to fully understand Ch cell innervation of pyramidal neurons. Additionally, it remains unclear whether there is complete loss of innervation to some pyramidal neurons in cases with autism, or if there remains an equivalent number of pyramidal neurons receiving input from Ch cell cartridges. If innervation is conserved in some pyramidal neurons but entirely lost in others, net inhibitory signaling could still be altered. Nevertheless, the data presented here do not provide evidence that synaptic contact between Ch cell axons and pyramidal neurons is altered in autism.

### The size of Ch cell boutons is decreased in autism

Ch cell terminal bouton size and innervation pattern are linked to synaptic transmission. Though the majority of terminal boutons contain a single synaptic junction, 5% of boutons analyzed in the rat frontal cortex contained two synaptic junctions [44]; while these findings mainly pertained to boutons with dendritic targets, it is feasible that decreased bouton size in autism may diminish the potential for double synapse formation. In addition, bouton size correlates to the amount of synaptic vesicles stored. Synaptic transmission depends on the continued availability of neurotransmitter-filled synaptic vesicles for triggered release from presynaptic boutons. Small boutons contain fewer synaptic vesicles [46], so it is possible that the reduced bouton size in autism reduces the strength of the inhibitory signal transmission of Ch cells. Bouton size is linked with axo-axonic inhibitory synapse activity. Studies performing knockdown of DOCK7 in Ch cells in the cortex resulted in a disorganized network of Ch cartridges and a reduction in the number and size of boutons, while ectopic expression of DOCK7 produced the opposite phenotypes [20]. DOCK7 affects Ch cell cartridge/bouton development by modulating the activity of ErbB4 [20,47,48]. ErbB4 is expressed by axon terminals in PV+ cells. Gain- and loss-of-function experiments demonstrated that ErbB4 promotes the formation of axo-axonic inhibitory synapses on pyramidal cells [47]. Interestingly, bouton size and activity are altered in schizophrenia in ways similar to our findings in autism [20,47].

## Limitations

As truncation of cartridges occurs when visualized in two dimensions and thus allowed analysis of only a handful of complete cartridges per field, three-dimensional analysis may add to our data. Using a larger sample size may also allow for more data to be gathered; however, the number of subjects with autism whose postmortem brain tissue is available for research remains very limited. In addition, decreased immunostaining may represent decreased PV expression in autism as opposed to a morphological change, but we think that is less likely since our previous work demonstrated that the decreased Ch cell number can be detected not only with PV but also with other markers such as GAT1.

## Conclusion

Our data suggest that a change in bouton density and/or cartridge length in surviving Ch cells are not factors associated with previously shown changes in the prefrontal cortex in autism. Instead, reduced bouton size along with the previously shown decreased number of Ch cells may impact the quantity and/or strength of inhibitory synapses in autism.
